# The effect of non‐absorbent hydrophobic sand litter on the urine protein‐to‐creatinine ratio in feline urine

**DOI:** 10.1111/vcp.13110

**Published:** 2022-04-01

**Authors:** Josh M. Kennils, Christina L. Maunder, Marta T. Costa

**Affiliations:** ^1^ The University of Bristol Bristol UK; ^2^ IDEXX Laboratories Limited Wetherby UK

**Keywords:** proteinuria, renal, urinalysis

## Abstract

**Background:**

Proteinuria can be quantified through the measurement of the urine protein‐to‐creatinine ratio (UPC). Voided urine samples in cats are often exposed to a non‐absorbable litter substrate prior to collection and urinalysis. Little is known about the effect exposure to such substrates has on pre‐analytical variability of UPC measurements.

**Objectives:**

The aim of this study was to assess agreement between UPC measurements from urine obtained by cystocentesis before and after exposure to non‐absorbent hydrophobic sand for 24 hours.

**Methods:**

UPCs were measured in 40 urine samples obtained by cystocentesis from 39 cats (baselineUPC). Urine was then exposed to non‐absorbent hydrophobic sand litter for 24 hours, recovered, and repeat UPCs were measured (litterUPC). Agreement between paired measurements and the presence of any bias or error was evaluated using Bland–Altman analysis Passing–Bablok regression analysis, respectively. Cohen's kappa was used to measure agreement for the International Renal Interest Society (IRIS) proteinuria classification of samples. Observed total error (TE_obs_) was calculated for the laboratory analyzer and compared against absolute percentage changes in paired UPC measurements.

**Results:**

Neither proportional nor constant error was identified using Passing‐Bablok regression between baselineUPC and litterUPC. Visual inspection of the Bland–Altman plot revealed good agreement, with 95% of paired measures falling within the limits of agreement (LOA). Cohen's kappa demonstrated almost perfect agreement for the IRIS classification of proteinuria between baselineUPC and litterUPC. Absolute percentage changes of paired UPC measurements outside of the LOAs were lower than the inter‐assay TE_obs_.

**Conclusions:**

Feline urine exposed to non‐absorbent hydrophobic sand litter appears acceptable for UPC measurements.

## 1 INTRODUCTION

Quantification of proteinuria can be achieved through the measurement of the urine protein‐to‐creatinine ratio (UPC). A single UPC measurement has been shown to correlate well with 24‐hour urinary protein loss in the cat, considered the gold standard for proteinuria quantification.^1^ The UPC is frequently measured as part of the diagnostic protocol and for prognostic purposes in several disease processes, including glomerulopathies, chronic kidney disease, and hypertension.^2^

Voided feline urine samples are often obtained using non‐absorbent cat litter substrates, such as polypropylene beads or hydrophobic sand.^3^ The use of hydrophobic sand has been shown to have no affect the measurement of urine specific gravity, urinary dipstick parameters (leukocytes, nitrites, urobilinogen, protein, pH, blood, USG, ketones, bilirubin, and glucose), and urinary creatinine and corticosterone concentrations in rats.^4^ A recent study documented that exposure to plastic non‐absorbent spherical pellets for 1 hour had no impact on feline UPC measurements.^5^ To the authors' knowledge, no information about the pre‐analytical effect of exposure to non‐absorbent hydrophobic sand litter on UPC measurements has been reported.

The analytical variability for feline urinary protein concentration measurements has been shown to vary considerably depending on which measurement method was used.^6^ The concept of total allowable error (TE_a_) allows the assessment of analytical performance. For veterinary species, the American Society of Veterinary Clinical Pathology (ASVCP) has defined TE_a_ for a number of biochemical analytes and electrolytes in serum.^7^ In human medicine, TE_a_ for analyte measurements is reported in the Clinical Laboratory Improvement Amendments (CLIA).^8^ The UPC is determined from the individual measurements of urinary protein and urinary creatinine concentrations, of which guidelines for TE_a_ have not been reported by the ASVCP or CLIA. Total observed error (TE_obs_) combines imprecision measures and bias and is unique to each analyzer and analyte. Where TE_obs_ < TE_a_, the analyzer performance is deemed acceptable for the analyte in question. Where TE_obs_ > TE_a_, investigations for causes of analyzer imprecision and bias are required.^7^ The aim of this study was to assess agreement between UPC measurements from urine obtained by cystocentesis before and after exposure to non‐absorbent hydrophobic sand for 24 hours using a method comparison study. Moreover, to further evaluate the significance of variability, percentage changes in paired UPC measurements were also compared with the analytical variability of the method. In the absence of a reported TE_a_ for UPC, we used TE_obs_ in this study as a measure of variability. It was hypothesized that there would be good agreement between paired UPC measurements.

## 2 METHODS AND MATERIALS

### 2.1 UPC comparison

Paired UPC measurements from 40 urine samples were obtained from 39 client‐owned cats at baseline and after 24 hours of contact with the non‐absorbent litter. Thirty‐four urine samples were collected by means of cystocentesis using ultrasound guidance at a referral hospital, and six were submitted to the Diagnostic Laboratory (Langford Vets, Bristol, UK) for routine urinalysis from first opinion veterinary practices and marked as urine samples obtained by cystocentesis between February 2019 and July 2019. Cats were included irrespective of age, sex, neuter status, health status, including recent drug administration or urine sediment status. Urine samples were collected as part of a wider study which was approved by the University of Bristol Research Ethics Committee (VIN/17/037).

Fifteen uncentrifuged whole urine samples were stored at 4°C until the time of the experiment and 25 uncentrifuged whole urine samples were stored at −80°C until the time of the experiment. Chilled samples were stored for no longer than 90 days and frozen samples for no longer than 120 days. Analyses were performed on two separate days. The 15 chilled samples were analyzed in one session and the frozen 25 samples in a second session. All samples were brought to room temperature and mixed by inversion prior to analysis.

For UPC measurements, 5 mL of urine was centrifuged in a conical tube at 438*g* for 5 minutes. The supernatant was used to determine the baseline UPC (baselineUPC). Urinary protein concentrations (UP) and urinary creatinine concentrations (UC) were determined using the pyrogallol red method and an enzymatic method, respectively, with an automated commercial analyzer (Reagents: U/CSF PROT and CreaE A, respectively, KoneLab 60i Prime, Thermo Fisher Scientific Ltd.). Urine supernatants were diluted 1:15 to determine UC. For UC determination, briefly, creatinine is converted to sarcosine, which is subsequently converted to glycine, formaldehyde, and hydrogen peroxide. Hydrogen peroxide reacts with 4‐aminophenazone and 2, 4, 6‐triiodo‐3‐hydroxybenzoic acid to produce a quinone imine chromogen. The color intensity of the chromogen is directly proportional to the initial concentration of creatinine present in the sample and is measured photometrically at 540 nm. The UPC was calculated as follows: urinary protein (mg/dL) ÷ urinary creatinine (mg/dL). Low‐ and high‐range urinary protein and urinary creatinine quality controls (uTrol and uTrol High, respectively, Thermo Scientific) were run twice daily. Calibration for urinary protein was performed every 14 days and for urinary creatinine every 7 days. If Westgard rule 1_2s_ was breached, calibration was performed earlier than scheduled and investigated as required, with samples and controls rerun; this was not required during the study. Paired UPC measurements were obtained within the same calibration run.

Up to 3 mL (range, 2.5–3.0 mL) of urine was placed into a Petri dish containing 4 g of non‐absorbent hydrophobic sand litter (Medicat, GlobalTech International Ltd.) using a pipette and covered, but not hermetically sealed. Petri dishes were left at room temperature (~20°C) for24 hours. Urine was recovered using a pipette, and repeat UPCs were measured (litterUPC).

### 2.2 Calculation of TE_obs_ for UPC

Observed total error for the methods routinely used for UPC measurements at the authors' institution was calculated by combining low‐ and high‐range quality control materials of urinary protein and urinary creatinine as supplied by the analyzer manufacturer. Intra‐assay and inter‐assay runs were performed in accordance with the laboratory quality control procedures. For the intra‐assay imprecision determination, urinary protein and creatinine concentrations were measured in the same respective quality control sample 20 times within one run consecutively. For inter‐assay imprecision determination, urinary protein and creatinine concentrations were measured from their respective quality control samples twice a day, in the same run, for 20 consecutive working days. The UPC was calculated from the measured protein and creatinine concentrations, and the ratio was used to determine TE_obs_.

### 2.3 Statistical analysis

#### 2.3.1 UPC comparison

Statistical analyses were performed using commercially available software packages (IBM SPSS Statistics for Windows version 24 and GraphPad Prism for Windows version 8). For all analyses, statistical significance was set at *P* < 0.05. All non‐categorical data were assessed for normality of distribution using the Shapiro–Wilk test and were found to be non‐Gaussian. Paired UP, UC, and UPC median values and ranges are reported, with median differences in measurements assessed using the Wilcoxon signed‐rank test. Mean changes for the standard deviation (SD) in UPC were calculated and reported as absolute values and absolute percentage changes. The correlation between the paired UP, UC, and UPC measurements was assessed using Spearman rank‐order correlation. The strength of correlation as determined by *r*_s_ was interpreted as follows: very high: 0.90–1.00, high: 0.70–0.80, moderate: 0.50–0.69, low: 0.30–0.49, little, if any: 0.00–0.29.^9^

Passing–Bablok regression was used to determine the presence of proportional or constant error between paired UP, UC, and UPC measurements. A Bland–Altman plot was used to calculate the mean bias and confer agreement between samples. If the 95% limits of agreement (LOA) spanned zero, no bias was evident. A subjectively small mean bias and subjectively narrow LOA as well as ≥95% of data points falling within the LOA suggested good agreement.^10^, ^11^ Cohen's kappa was used to measure agreement between International Renal Interest Society (IRIS) categories for proteinuria between baselineUPC and litterUPC. Categories of feline proteinuria according to IRIS are as follows: UPC < 0.2, non‐proteinuric, UPC 0.2 to 0.4, borderline proteinuric, and UPC > 0.4, proteinuric.^12^ The strength of agreement as determined by *κ* was interpreted as follows: almost perfect: 0.81–1.00, substantial: 0.61–0.80, moderate: 0.41–0.60, fair: 0.21–0.40, slight: 0.00–0.20 and poor: <0.00.^13^

#### 2.3.2 Calculation of TE_obs_ for UPC

For both low‐ and high‐range UPC, the mean, SD, bias, coefficient of variation (CV), and TE_obs_ were calculated. Bias(%) was calculated as follows: (Target UPC from controls – mean UPC) ÷ mean UPC × 100. The CV (%) was calculated as follows: SD ÷ mean x 100. Total observed error (%) was calculated as follows: 2CV + absolute bias. TE_obs_ was used to compare paired UPC measurements.^7^ If the absolute percentage change between paired measurements differed by more than TE_obs_, then this difference was considered to be due to more than analytical variability and, therefore, of possible significance.

## 3 RESULTS

### 3.1 UPC comparison

Median baselineUP and baselineUC were 38.13 mg/dL (range, 7.35–277.9 mg/dL) and 215.58 mg/dL (range, 17.54–640.21 mg/dL), respectively. Median litterUP and litterUC were 37.85 mg/dL (range, 5.96‐279 mg/dL) and 215.67 mg/dL (range, 18.10–657.01 mg/dL), respectively. The median decrease in UP and median increase in UC observed were statistically significant (*P* = 0.03 and *P* < 0.0005, respectively). Median baselineUPC was 0.17 (range, 0.05–5.59) and the median litterUPC was 0.16 (range, 0.05–5.52). A decrease in UPC was observed at 24 hours in 29 cases, an increase in UPC was observed in three cases, and no change in UPC was observed in eight cases. There was a statistically significant median decrease in UPC of 0.01 after urine was exposed to non‐absorbent hydrophobic sand litter for 24 hours (*P* < 0.0005).

The mean absolute change between the baselineUPC and litterUPC was 0.04 (SD 0.08), equivalent to a 9.08% (SD 8.37%) mean absolute percentage change. Absolute percentage changes between paired measurements ranged from 0% to 31.38%.

**FIGURE 1:**
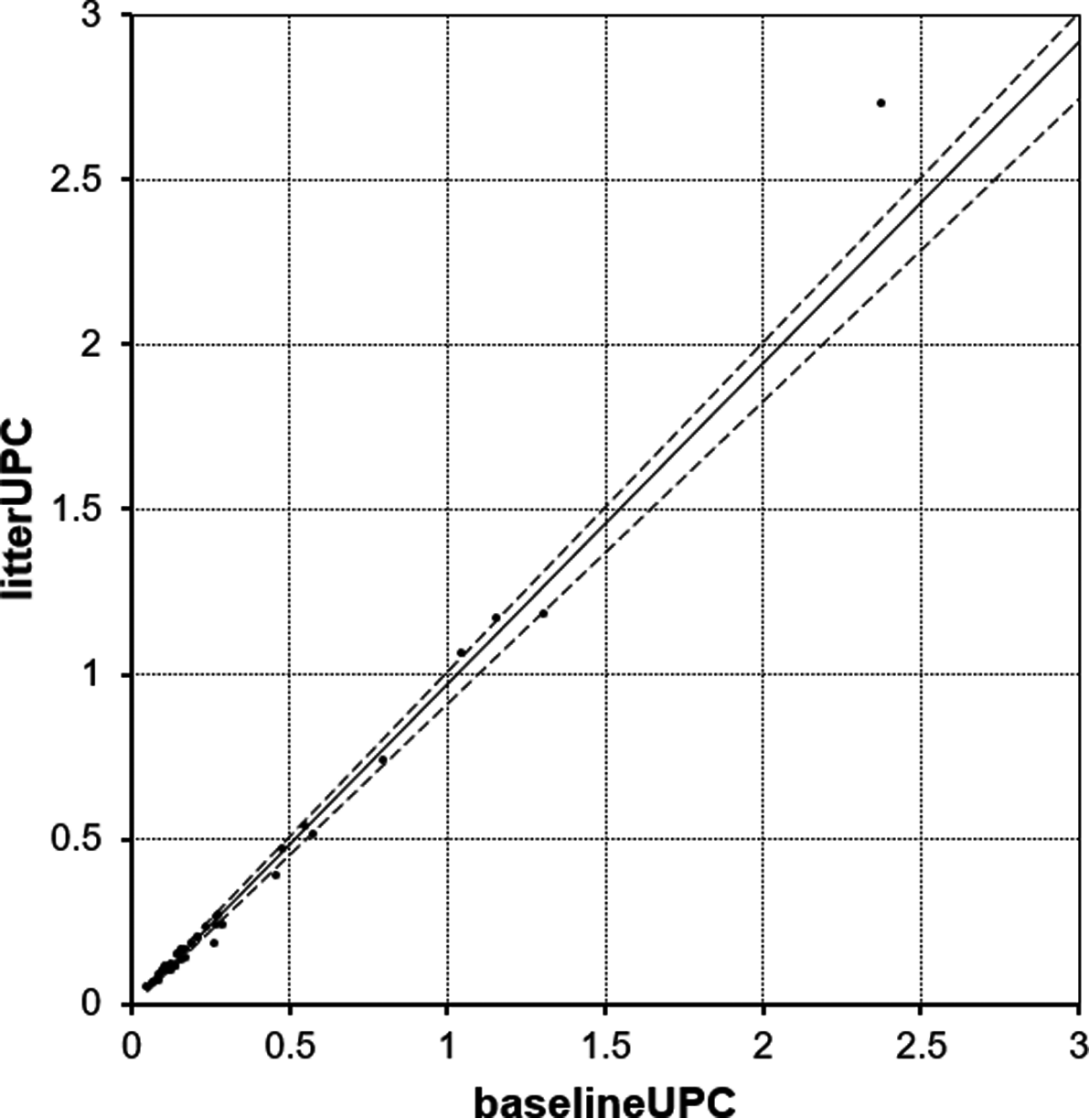
Passing–Bablok plot of urine protein‐to‐creatinine (UPC) measurements obtained at baseline (baselineUPC), and those obtained after 24‐h exposure to non‐absorbent hydrophobic sand litter (litterUPC) in 40 samples; The solid line is the regression line (*y* = −0.0038 + 0.9606x), and the dotted lines are the 95%CI. One point (baselineUPC = 5.59, litterUPC = 5.22) was omitted to allow for better visualization of lower UPC values

**FIGURE 2:**
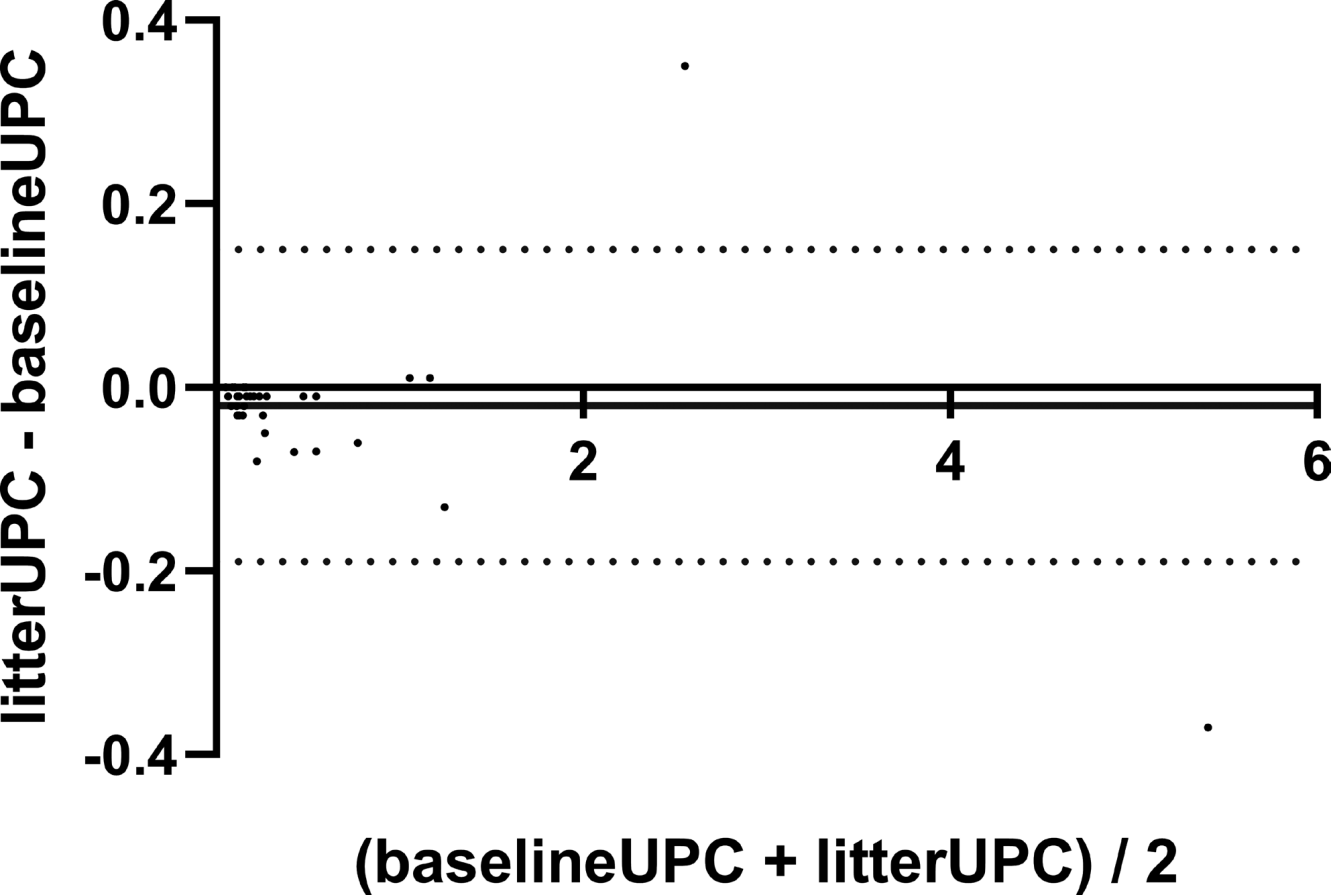
Difference vs average Bland–Altman plots of urine protein‐to‐creatinine (UPC) measurements obtained at baseline (baselineUPC) and after 24‐h exposure with non‐absorbent hydrophobic sand litter (litterUPC) in 40 samples. Solid line represents the mean of the difference (bias) = −0.02075. Dashed lines represent the 95% limits of agreement from −0.1896 to 0.1481

Table 1 presents the correlation (*r*_s_), slope and intercept for the Passing‐Bablok analysis and mean bias with LOA for Bland–Altman analyses for UP, UC, and UPC. The Passing–Bablok regression analysis revealed neither proportional nor constant error with confidence intervals, including 0 and 1, respectively, for UPC (Figure 1). The difference vs the average Bland–Altman plot revealed good agreement between paired UPC measurements (Figure 2). All but two differences in UPC measurements fell within the Bland–Altman LOA. Of the two that did not, actual UPC values decreased from 5.59 to 5.52 and increased from 2.38 to 2.73, equivalent to the absolute percentage changes of 6.62% and 14.71%, respectively.

**TABLE 1:** Spearman correlation (*r*_s_), slope, and intercept with 95% confidence intervals (CI) of Passing–Bablok and Bland–Altman mean bias with 95% limits of agreement (LOA) for urinary protein (UP), urinary creatinine (UC) and urine protein‐to‐creatinine ratio (UPC) measured at baseline vs UP, UC, and UPC measured after 24‐h exposure with non‐absorbent hydrophobic sand litter

	Spearman correlation	Passing–Bablok		Bland–Altman
	*r*_s_	Slope (95% CI)	Intercept (95% CI)	Mean Bias (lower LOA to upper LOA)
UP	0.99	1.02 (1.00 to 1.06)	−1.88 (−3.34 to −1.20)	−0.12 (−9.18 to 8.94)
UC	0.99	1.02 (1.00 to 1.05)	0.08 (−2.15 to 3.31)	13.61 (−29.24 to 56.45)
UPC	0.99	0.96 (0.92 to 1.00)	0.00 (−0.01 to 0.00)	−0.02 (−0.19 to 0.15)

The number of urine samples classified as non‐proteinuric, borderline proteinuric, or proteinuric for baselineUPC and litterUPC is reported in Table 2. Categories for proteinuria for baselineUPC and litterUPC were concordant in 38 cases. Of the two cases that changed a category, one went from borderline proteinuric to non‐proteinuric, and one went from proteinuric to borderline proteinuric. The IRIS proteinuria category changed by no more than one level for any discordant case. The kappa coefficient was *κ* = 0.915, corresponding to almost perfect agreement.^13^

**TABLE 2:** Number of samples within each International Renal Interest Society (IRIS) proteinuria category at baseline and at 24 h. Discordant results are highlighted

		IRIS proteinuria category at 24 h		
		Non‐proteinuric	Borderline proteinuric	Proteinuric
IRIS proteinuria category at baseline	Non‐proteinuric	22	0	0
	Borderline proteinuric	1	7	0
	Proteinuric	0	1	9

### 3.2 Calculation of TE_obs_ for UPC

Low‐ and high‐quality control targets for UP, UC, and subsequently calculated UPC targets with the intra‐ and inter‐assay mean measures, SD, bias, CV, and TE_obs_, are reported in Table 3. The absolute percentage changes between 35 paired UPC measurements were below the high‐range inter‐assay TE_obs_; the absolute percentage changes of 5 paired UPC measurements were above the high‐range inter‐assay TE_obs_. The absolute percentage change for 1 paired UPC measurement was above the low‐range inter‐assay TE_obs_.

**TABLE 3:** The mean, standard deviation (SD), bias, coefficient of variation (CV), and observed total error (TE_obs_) for intra‐ and inter‐assay runs of low‐ and high‐range UPC controls calculated from quality control materials (QCM) for urinary protein (UP) and urinary creatinine (UC) concentrations

UPC target	QCM		UPC	Intra‐assay					Inter‐assay				
	UP (mg/dL)	UC (mg/dL)		Mean	SD	Bias	CV%	TE_obs_ (%)	Mean	SD	Bias	CV%	TE_obs_ (%)
Low‐range	8	78.05	0.10	0.10	0.01	3.0	5.9	14.83	0.11	0.01	−6.4	29.4	29.39
High‐range	70	177.60	0.39	0.39	0.01	2.3	2.0	6.36	0.37	0.02	6.1	18.9	18.87

## 4 DISCUSSION

The main objective of this study was to evaluate what effect if any, 24‐hour exposure to non‐absorbent hydrophobic sand litter had on UPC measurements in feline urine. This was achieved through means of a method comparison study which demonstrated good agreement between baselineUPC and litterUPC. Although statistically significant changes in median UP, UC, and UPC were observed, the subsequent measures of agreement demonstrate that exposure of urine to non‐absorbent hydrophobic sand litter is unlikely to affect clinical decision‐making. This is particularly highlighted by an almost perfect agreement in IRIS categories of proteinuria between the baselineUPC and litterUPC.^13^ In our study, only two paired samples out of 40 changed IRIS category; one from borderline proteinuric to non‐proteinuric (UPC: 0.26 to 0.18) and one from proteinuric to borderline proteinuric (UPC: 0.46 to 0.39). Absolute percentage changes for these cases were 31.38% and 15.22%, respectively. The former is just above our low range inter‐assay TE_obs_ and so possibly reflects more than analytical variability in this case; the latter is below our high range inter‐assay TE_obs_, likely merely reflecting analytical variability. This finding could allow for serial UPC measurements in cats to be obtained by owners in the home environment. Collection at home would have significant cost benefits to owners and might also reduce stress for feline patients. Our findings are in agreement with a recent study by Giraldi and others, which demonstrated that urine exposure to non‐absorbent plastic bead litter for 1 hour had no effect on feline UPC measurements.^5^ However, our study evaluated the effect of a longer duration of exposure (24 hours) to better mimic what may happen in the home environment, where litter trays may only be emptied once a day. We also used an alternative litter substrate, included 40 samples, as the suggested requirement for a method comparison study, and used a different approach to assess variability between baselineUPC and litterUPC in the absence of TE_a_ for UPC measurements, further supporting that using hydrophobic sand for collection of urine samples does not seem to contribute to preanalytical variability of the UPC measurement.^14^

As this study was purely analytical in nature, no exclusions were applied with regard to age, sex, neuter status, health status, including recent drug administration, or urine sediment status. In human medicine, the presence of aminoglycosides in a sample has been shown to interfere with the pyrogallol red assay.^15^ This has yet to be evaluated in cats specifically, but no cats in this study had a history of recent aminoglycoside administration. The presence of an active sediment has been shown to influence UPC measurements.^16^, ^17^ Our aim was to evaluate variability between baselineUPC and litterUPC; to encompass a broader range of UPC measurements, we included samples with active sediments in this study. Although urine samples were stored under different conditions, which may have affected UPC measurements, this study only compared the difference in UPC measurements taken 24 hours apart. Moyle and others showed that storage of urine at room temperature for up to 5 days did not affect UPC measurements in both proteinuric and non‐proteinuric dogs, and Giraldi and others showed that storage of urine at room temperature for 6 hours did not affect UPC measurements in cats.^5^, ^18^ In this study, all samples were brought to room temperature before baselineUPCs were measured. Samples were then exposed to the hydrophobic sand litter for 24 hours before the UPCs were re‐measured and so the initially different storage conditions are unlikely to have affected the results.

A limitation to this study is the use of TE_obs_ of the analyzer as guidelines for an acceptable degree of change due to the lack of TE_a_ guidelines for UPC measurements. Total observed error was calculated for low and high range UPC values of 0.10 and 0.39, respectively, as determined by the quality control materials for urinary protein and urinary creatinine concentrations. These values are non‐proteinuric and borderline proteinuric, respectively, by IRIS, which did not reflect the nature of all our samples. The high range UPC value is close to the IRIS‐defined threshold for proteinuria in cats (>0.4). The inter‐assay TE_obs_ at this range suggests that there might be a low risk of borderline proteinuric samples being categorized as proteinuric and vice versa, as was seen in this study. It would be of interest to calculate TE_obs_ for UPC values of higher magnitude. However, for the two differences in UPCs that fell outside the Bland–Altman plot LOAs, the absolute percentage changes of 6.62% and 14.71% are below the inter‐assay TE_obs_ for high range UPCs of 18.87%, suggesting analytical variability alone could account for the differences in measurements observed in these samples. The calculated CV for the intra‐assay low‐range UPC of 5.9% was of a similar magnitude to the imprecision observed in a study by Giraldi and others.^6^ The low‐range inter‐assay CV in this study of 29.4% was much higher than the 16.4% reported by Giraldi and others.^6^ The reason for such a difference is likely multifactorial, including differences in sample handling, laboratory protocols, and analyzers used (ie, variation in analyzer performance at low or high analyte concentrations). Rossi and others have previously reported inter‐laboratory variation in UPC measurements, and it is for these reasons that serial UPC measurements should be obtained through the same laboratory.^19^ The magnitude of both the low‐ and high‐range inter‐assay CV in this study and in the study by Giraldi and others highlights the need for further similar studies at higher UPC ranges (ie, proteinuric ranges that would warrant anti‐proteinuric therapy).^6^ In dogs, it has been suggested that when treating proteinuria, a difference of up to 80% in serial measurements should be used as a target, particularly at low UPC ranges, to overcome the inherent imprecision in UPC measurements and the day‐to‐day variation in urinary protein excretion.^20^

In this study, repeat UPC measurements were obtained after 24 hours of exposure to hydrophobic sand litter. In the clinical setting, it is unlikely urine would be exposed to this litter substrate for a longer duration, but further studies are needed to evaluate the effect of longer duration of exposure. This study also only evaluated one type of non‐absorbent litter. Other non‐absorbent materials, such as non‐absorbent plastic pellets, are commonly used as a non‐invasive method of urine collection in the cat, but thus far, only exposure to this substrate for 1 hour has been evaluated.^3^, ^5^

In conclusion, both agreement measures of the Bland–Altman analysis and Cohen's kappa for IRIS proteinuria classification suggest that the use of non‐absorbent hydrophobic sand litter has minimal effect on UPC measurements; and therefore, feline urine exposed to this litter substrate for up to 24 hours appears acceptable for UPC measurements.

## DECLARATIONS

Parts of this study's data formed part of the first author's Masters by Research thesis examined in February 2020. Parts of this study's data were presented in poster format at BSAVA Congress 2021.
